# Multiple splenic hamartomas and familial adenomatous polyposis: a case report and review of the literature

**DOI:** 10.1186/s13256-015-0627-3

**Published:** 2015-07-04

**Authors:** Nicola Carlomagno, Francesca Duraturo, Maria Candida, Marina De Rosa, Valeria Varone, Giuseppe Ciancia, Armando Calogero, Michele L Santangelo

**Affiliations:** 1 General Surgery Unit – Advanced Biomedical Science Department, University Federico II of Naples, Via S. Pansini 5, 80131 Naples, Italy; 2 Molecular Medicine and Medical Biotechnology Department, University Federico II of Naples, Naples, Italy; 3 Advanced Biomedical Science Department, University Federico II of Naples, Naples, Italy

**Keywords:** Familial adenomatous polyposis, Splenectomy, Splenic hamartomas

## Abstract

**Introduction:**

Splenoma or splenic hamartoma is a rare primary splenic tumor most often discovered radiologically and incidentally. Splenic hamartomas have a strong association with solid and hematological malignancies and, in rare cases, with tuberous sclerosis, but to the best of our knowledge no reports of splenic hamartomas associated with familial adenomatous polyposis have been documented, although it is recognized that familial adenomatous polyposis presents a variety of extracolonic manifestations.

**Case presentation:**

We report on a very rare case of multiple splenic hamartomas in a 46-year-old white woman who had previously undergone surgery for restorative proctocolectomy for familial adenomatous polyposis. A computed tomography scan of her spleen revealed multiple small lesions which measured less than 1cm in diameter. A splenectomy was performed and a histologic examination of the splenectomy specimen revealed the presence of multiple hamartomas.

**Conclusion:**

Incidence, differential diagnosis, diagnostic procedures, pathologic findings and treatment of splenic hamartomas are discussed here and hamartomas are considered in a differential diagnosis of splenic tumors. A splenectomy is indicated in cases where malignancy cannot be excluded and in cases of associated hematologic disorders. To the best of our knowledge our patient is the first reported case to have splenic hamartomas identified in a familial adenomatous polyposis-affected patient with mutation in exon 15 of the *APC* gene. At this time it is not possible to correlate with certainty our multiple splenic hamartomas and familial adenomatous polyposis case as a clinical manifestation of the mutation of *APC* gene; however, we believe that this case report could be important for further observation of similar cases in the future.

**Electronic supplementary material:**

The online version of this article (doi:10.1186/s13256-015-0627-3) contains supplementary material, which is available to authorized users.

## Introduction

Splenic hamartomas (SHs), also known as splenomas, accessory spleens in the spleen, congenital malformation and hyperplastic nodules, are rare benign vascular proliferative tumors with an anomalous mixture of normal splenic elements such as red and white pulp, and are characterized by cluster of differentiation (CD) 8 immunopositivity of the vascular endothelial lining cells [1–3].

Their diagnosis is often incidental at autopsy or when evaluating images for other reasons [1].

Although benign, it is imperative that a differential diagnosis be made between splenic primary and secondary malignancies [1, 4].

Here we report on a rare case of multiple SHs incidentally found in a woman who had previously been operated on for familial adenomatous polyposis (FAP) coli.

## Case presentation

**Fig. 1:**
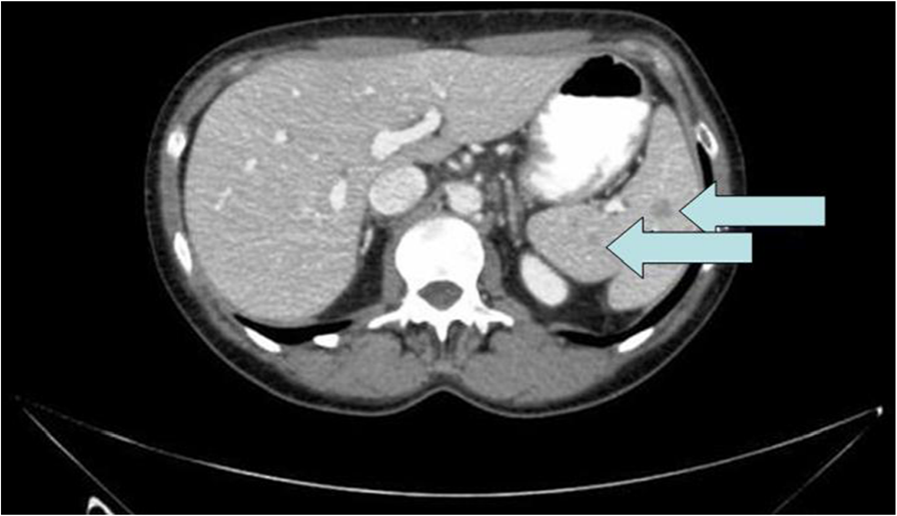
Dysmorphic and inhomogeneous spleen, with nodular millimeter areas – indicated with the arrows - which appeared hypodense during arterial and portal phases of the contrastographic study (transverse diameter max 11mm at abdominal CT scan)

A 46-year-old white woman was admitted for the presence of small multiple splenic lesions which were incidentally found by a routine abdominal computed tomography (CT) scan. At the scan she was found to have an incisional hernia at the midline incision of the previous colectomy. She was asymptomatic with unremarkable laboratory findings. On physical examination, no palpable mass in her abdomen was found. Upper and lower endoscopy and abdominal ultrasonography (US) did not show primary malignancies, while the CT scan (Fig. 1) revealed a dysmorphic and inhomogeneous spleen with nodular millimeter hypodense areas which were found to be almost isodense to the remaining splenic parenchyma in basic and later contrastographic scans (Additional file 1).

She had a 20-year history of FAP of a classic phenotype with profuse colorectal polyposis. She had successively undergone a restorative proctocolectomy (1995), exeresis of cranial osteomas (2003), hysteroannessectomy for a benign ovarian cyst and uterine myomatosis (2007), and has been regularly followed up. She also presented congenital hypertrophy of retinal pigment epithelium (CHRPE) and dental abnormalities. On mutational analysis, a mutation of the adenomatous polyposis coli (*APC*) gene in fragment C of exon 15, deletion of an A nucleotide (c.2638delA) was found which created a premature stop codon 105 nucleotides downstream and caused a truncated protein (p.Ile880SerfsX36).

A few days following the scan, she underwent a laparotomy with splenectomy and incisional hernia repair.

Her postoperative course was uneventful and she was discharged on the seventh postsurgical day.

**Fig. 2:**
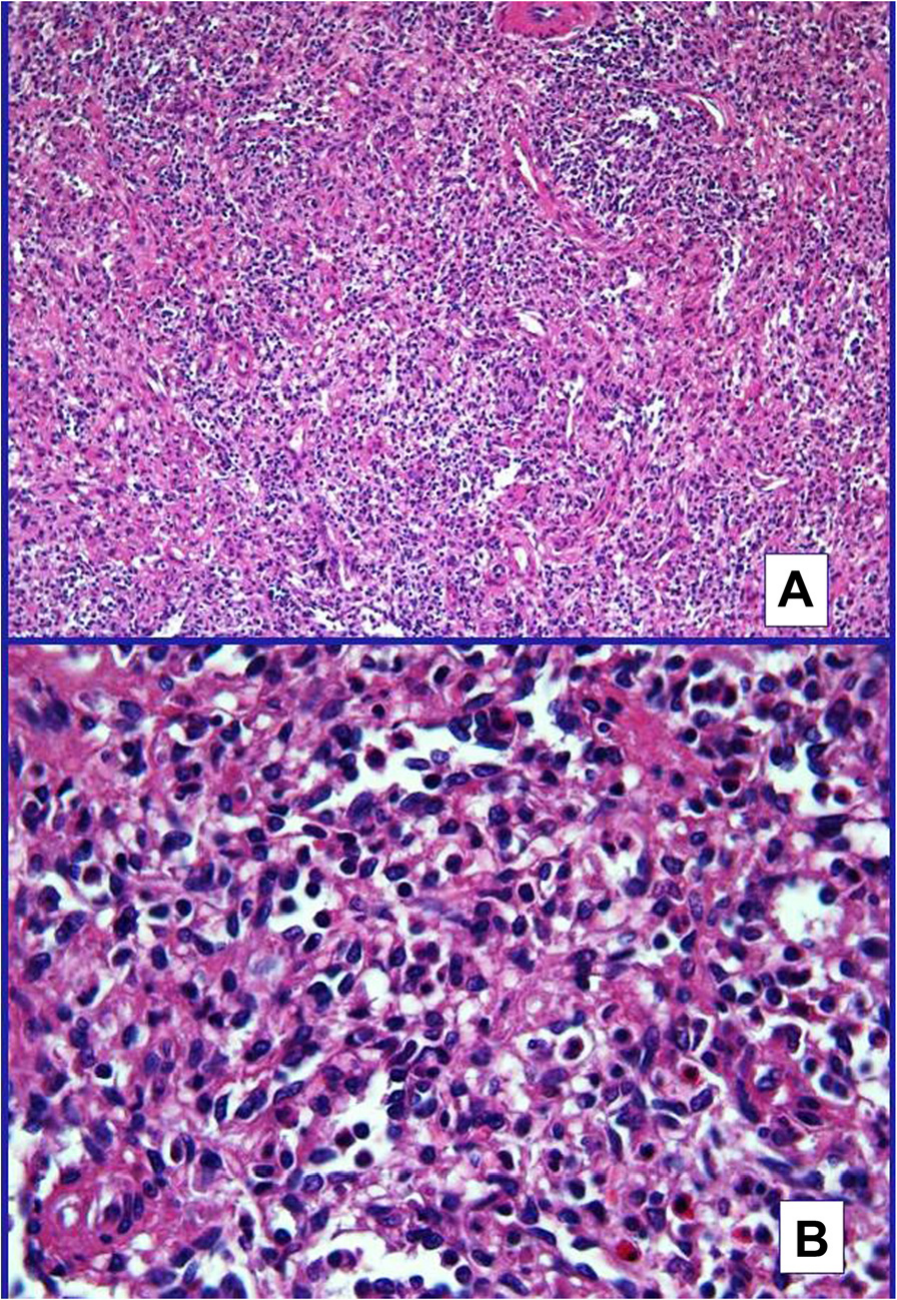
**a** Haphazardly arranged slit-like vascular process without intervening white pulp. Hematoxylin and eosin staining, ×200. **b** Vascular spaces lined by plump endothelial cells without atypia and containing red blood cells. Hematoxylin and eosin staining, ×400

**Fig. 3:**
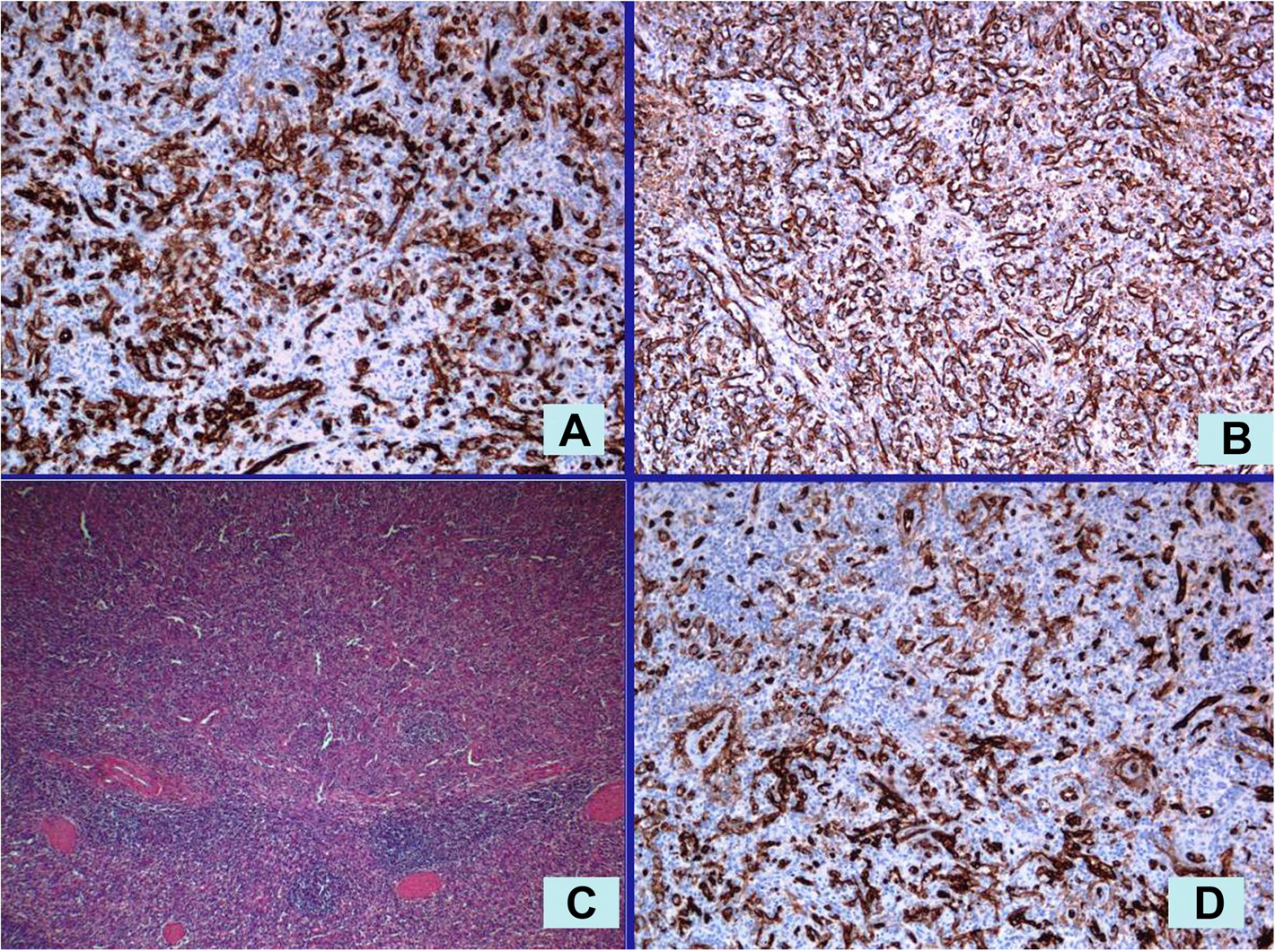
**c** The nodule was composed of disorganized red pulp elements and was separated from the normal spleen by a compressed rim of tissue and not by a true capsule or fibrosis. Hematoxylin and eosin staining, ×100. The endothelial cells lining the vascular spaces are positive for: **a** CD8, **b** vimentin, and **d** CD31. Immunohistochemical staining, ×100

Histological examination of the specimen (Figs. 2 and 3) revealed SHs.

The resected spleen measured 10×8×2cm and, on the cut section, showed multiple well-circumscribed bulging nodules ranging in size from 0.5cm to 1cm. These were red-tan, quite homogeneous and compressing the surrounding splenic parenchyma. On microscopic examination, the lesions were composed of disorganized cords and sinuses of red pulp with slit-like vascular spaces lined by plump endothelial cells containing erythrocytes and, occasionally, an amorphous material largely composed of trapped platelets. White pulp follicles were absent and only a scant trabecular structure was observed. No cytological atypia was observed. Although grossly well demarcated, the nodules were not capsulated, but surrounded by a rim of compressed red pulp separating them from the normal spleen without associated fibrosis. The residual spleen was histologically unremarkable.

On immunohistochemical examination, the endothelial cells lining the vascular channel were positive for CD8, CD31, CD34 and vimentin. The definitive diagnosis was multiple hamartomas of the spleen.

After the operation at a six-month follow-up, she was in good general health.

**Fig. 4:**
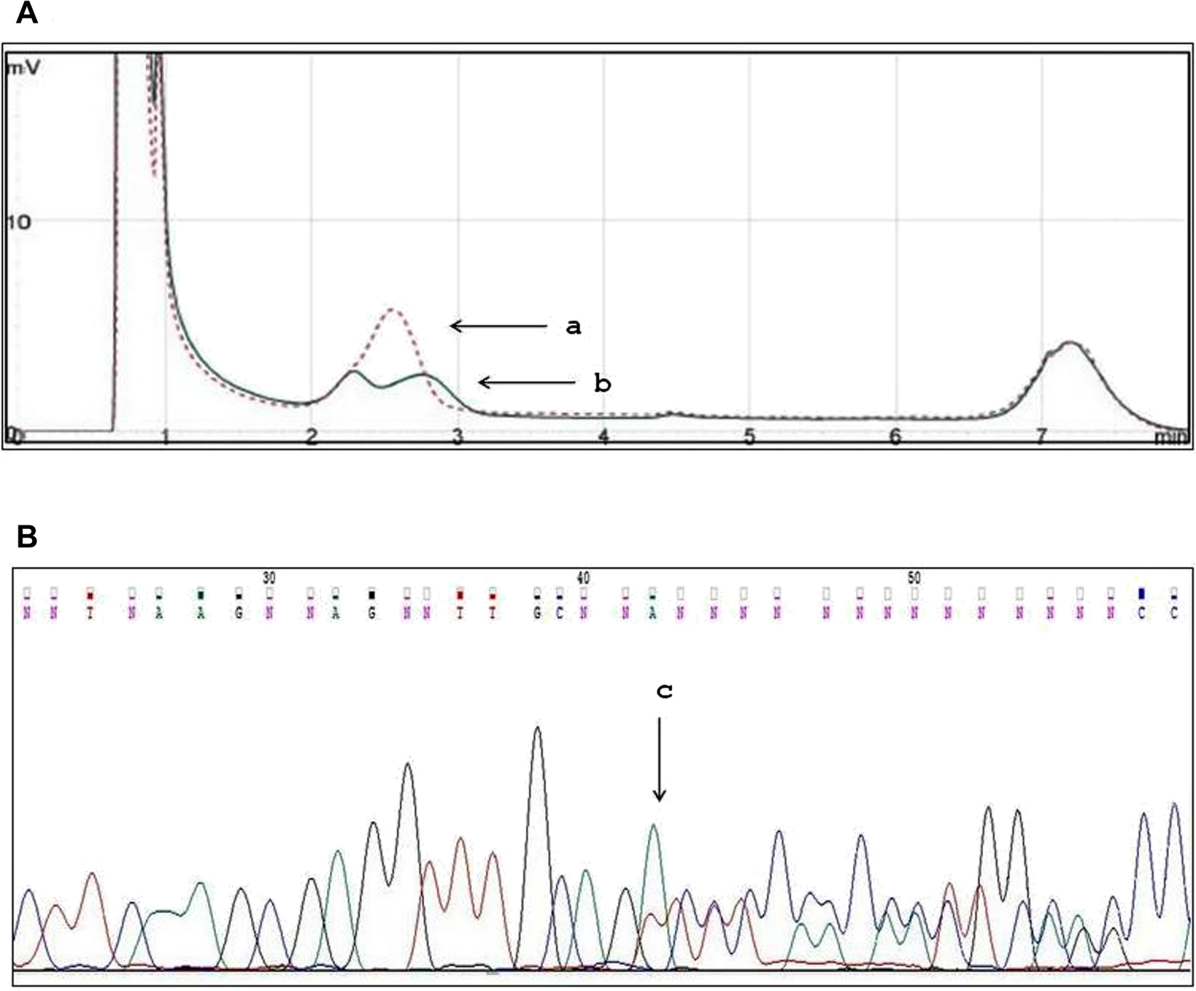
c.2638delA *APC* mutation detection on DNA extracted from a paraffin-embedded specimen of the splenic hamartoma. **a** Denaturing high-performance liquid chromatography and **b** sequencing analysis of amplified product corresponding to DNA region including the mutation c.2638delA of *APC* gene. *a* is carrier patient; *b* is normal control; *c* is the position of mutation, c.2638delA (the forward sequence is shown)

Subsequent to further analysis, we have confirmed the presence of the heterozygous mutation c2638delA in exon 15 of the *APC* gene, also on the DNA extracted from a paraffin-embedded specimen of the SHs (Fig. 4).

## Discussion

This rare case of multiple SHs with FAP led us to review the literature and to considerations on incidence, diagnosis, pathologic findings and treatment of SHs, in order to try to understand their likely association with FAP. While splenic tumors are uncommon, benign ones are extraordinarily rare (7/100,000 autopsy specimens) [5]. Rokitansky [6] first described a SH under the appellation of splenomas; since then, more than 150 cases have been reported with a prevalence of 0.024 to 0.13 % given in a review of autopsies [7] and three in 200,000 splenectomies [3].

The origin of SH is not clear with some hypotheses including congenital malformations of the splenic red pulp, neoplasms of the splenic red pulp, post-traumatic reactive lesions [3, 8] or acquired proliferative processes as documented in cases associated with hematological malignancies [4].

SHs may be associated with solid and hematological malignancies (that is, thymomas, squamous cell carcinomas and renal cell carcinomas) and in rare cases with tuberous sclerosis, an autosomal dominant disorder characterized by the development of benign tumors (hamartomas) in multiple organs (that is, brain, skin, lungs and kidneys) [1, 2].

Our case concerns a woman who received a molecular diagnosis of FAP. FAP is a dominant inherited autosomal disorder associated with mutations in the *APC* gene. In addition, bi-allelic mutations in the *MYH* gene may account for up to 30 % of families with multiple adenomas (15–100) which do not exhibit an autosomal dominant pattern of inheritance nor a germline mutation in the *APC* gene [9]. A variety of extracolonic manifestations (ECM) occur in patients with FAP and many of them could correlate to a specific mutation of the *APC* gene [10] (Table 1).

**Table 1:** Genotype/Phenotype correlations of extracolonic manifestations in patients with familial adenomatous polyposis and attenuated familial adenomatous polyposis

Phenotype	Exon of *APC* gene Codon position
Papillary thyroid carcinoma	3–15 exons Codons 140–1309
Hepatoblastoma	3–15 exons Codons 141–1578
Congenital hypertrophy of retinal pigment epithelium (CHRPE)	10–15 exons Codons 463–1445
Osteomas	15 exon Codons 767–1578
Desmoids	15 exon Codons 1309–1580

In a review of the medical literature, no reported association between FAP and SHs has yet been made. In addition, in hamartomatous polyposis syndromes, such as Peutz–Jeghers syndrome (PJS) an autosomal dominant syndrome arising from germline mutations in the serine threonine kinase gene (*STK11*) [11], SHs have not been identified although an exceptionally high rate of extracolonic cancers in patients with PJS including gastric (29 %), small bowel (13 %), pancreatic (36 %), breast (54 %), ovarian (21 %), lung (15 %), cervical (10 %), and uterine/testicular (9 %) [12] and many other benign lesions [13] were reported.

Nor was SH reported among numerous clinical manifestations [14] of hamartoma tumor syndrome, an autosomal disorder associated with germline mutation or a differential expression of phosphatase and tensin homolog gene (*PTEN*) on chromosome ten [15, 16].

In our patient a profuse FAP was diagnosed back in 1995 and a restorative proctocolectomy was performed. She presented several ECM: osteoma, dental anomalies, CHRPE, ovarian cysts and uterine myomatosis. At molecular analysis a mutation of the *APC* gene [17] was found in fragment C of exon 15, the deletion of an A nucleotide (c.2638delA) which determined a frameshift and therefore a premature stop codon, 105 nucleotides downstream causing the truncated protein (p.Ile880SerfsX36). This mutation was also identified in heterozygous on DNA extracted from the paraffin-embedded specimen of the SH. As expected, loss of heterozygosity (LOH) of the *APC* gene was not present and was consistent with the benign nature of the hamartomatous tumor [18].

Moreover, all carriers in her family presented a classic FAP phenotype with profuse polyposis of the colorectum and had undergone restorative proctocolectomies in the third decade of their lives. Mutations in exon 15 are common in affected patients with FAP and are associated with profuse polyposis and some ECM.

A correlation between the mutation site and clinical phenotype encompassing both colonic and repeatedly extracolonic features has been reported [19, 20]. Mutations at codon 1309 lead to the most severe intestinal phenotype characterized by a high number and early development of colorectal adenomas. Therefore it is likely that SHs are included among extracolonic manifestations.

SHs vary in size and sometimes can reach 20cm in diameter. Two histological types exist according to the tumor components [3]: a white pulp type and a red pulp type. The white pulp type is composed entirely of lymphoid tissue, while the red pulp type is composed of an aberrant complex of sinuses. Most tumors are a mixture of the two subtypes. Their characteristic histological features are disorganized vascular channels lined with plump endothelial cells. The lining cells of the vascular channels are typically positive for CD8, CD31, CD34, factor-related antigen and vimentin [1, 2, 21]. Immunohistochemical staining may distinguish a SH from a capillary hemangioma according to their respective staining characteristics. Endothelial cells which are positive for CD8 are a key feature that distinguishes a hamartoma from other vascular lesions of the spleen [1, 21, 22]. The endothelial cells of SHs are CD8-positive in contrast with the CD8-negative of hemangiomas [22].

As in our case, the majority of patients (80 %) with hamartomas do not have any symptoms and an incidental diagnosis is made during autopsy or an image evaluation [2]. In the other cases splenomegaly, palpable mass, spontaneous rupture of the spleen, anemia, thrombocytopenia and digestive symptoms may allow for identification [1, 23].

The diagnosis of hamartomas can be a challenge as it is difficult to distinguish them from splenic primary malignancies with imaging modalities [22]. CT scans and US features have been highly variable and nonspecific. Recently, the radiological features on US, color Doppler imaging, CT and magnetic resonance imaging (MRI) have been able to clearly identify hamartomas [23]. Such tumors may appear hyperechoic on US, or as isodense or hypodense solid masses relative to the adjacent normal splenic parenchyma on CT. On color Doppler the blood flow results are increased due to hypervascularity.

The main differential diagnosis includes other vascular tumors and solid mass-forming lesions (hemangioma, littoral cell angioma, lymphangioma, hemangioendothelioma, sclerosing angiomatoid nodular transformation, angiosarcoma inflammatory myofibroblastic tumor, lymphoma and metastatic tumors) [1, 23]. Once identified, a pathological examination is strongly needed. The risk of bleeding or tumor dissemination makes a fine needle aspiration biopsy of the spleen problematic [22] and therefore surgery is necessary for diagnostic and therapeutic purposes.

Once a splenic lesion is discovered, a malignant tumor cannot be ruled out without performing a splenectomy. A splenectomy may be curative even for hematological disorders associated with hamartomas. Although recently laparoscopic surgery has become the standard technique for splenic disorders, due to our patient’s previous colectomy, we adopted a laparotomic approach due to the presence of several tenacious intraperitoneal adherences.

## Conclusions

SHs are benign vascular proliferative lesions with a characteristic CD8-positive immunophenotype of the lining endothelial cells. A splenectomy is mandatory to exclude primary or secondary malignancies. To the best of our knowledge, this is the first reported SH case identified in a FAP-affected patient with mutation in exon 15 of the *APC* gene. We therefore feel that the topic of this report could be relevant for further observation of similar cases in the future.

## Consent

Written informed consent was obtained from the patient for publication of this case report and any accompanying images. A copy of the written consent is available for review by the Editor-in-Chief of this journal.

## Additional file

Additional file 1: **46 years old FAP affected patient with splenic hamartomas: main steps of her clinical history.**

## Abbreviations

*APC*: Adenomatous polyposis coli
CD: Cluster of differentiation
CHRPE: Congenital hypertrophy of retinal pigment epithelium
CT: Computed tomography
ECM: Extracolonic manifestations
FAP: Familial adenomatous polyposis
PJS: Peutz–Jeghers syndrome
SH: Splenic hamartoma
US: Ultrasonography
