# Supplementary material for: Multiple splenic hamartomas and familial adenomatous polyposis: a case report and review of the literature
Source: J Med Case Rep. 2015 Jul 4;9:154. doi: 10.1186/s13256-015-0627-3 (PMC4507323; doi:10.1186/s13256-015-0627-3)
Supplement: Additional file 1: — 46 years old FAP affected patient with splenic hamartomas: main steps of her clinical history. [file 13256_2015_627_MOESM1_ESM.doc]

Additional file 1 –

**46 y.o FAP affected patient with splenic hamartomas :**

**main steps of her clinical history**

Lab: unremarkable laboratory findings

Past illness:

20 year history of FAP of a classic phenotype with profuse colo-rectal polyposis

mutation of the APC gene in fragment C of exon 15, deletion of an A nucleotide (c.2638delA)

History

incisional hernia at the midline incision of the previous colectomy. Asymptomatic

Upon physical examination, no palpable mass in the abdomen

Upper and lower endoscopy and abdominal ultrasonography negative

**Presence**

**of genetic Birth in 1968 Past history Presenting concerns Diagnosis Intervention Post-operative course Follow-up**

**history**

**absence of**

restorative proctocolectomy (1995), exeresis of cranial osteomas (2003), hysteroannessectomy for a benign ovarian cyst and uterine myomatosis (2007),

At CT scan: dysmorphic and inhomogeneous spleen with nodular millimeter hypodense areas which were found to be almost isodense to the remaining splenic parenchyma in basic and later contrastographic scans

**environmental**

splenectomy and incisional hernia repair

Uneventful

at a 6th month follow up in good general health

**and**

**lifestyle**

**influences**

discharged on the 7^th^ post-surgical day

Histology:

multiple hamartomas of the spleen Immunohistochemically

endothelial cells lining the vascular channel were positive for CD8, CD31, CD34 and vimentin.

heterozygous mutation c2638delA in exon 15 of the APC gene, also on the DNA extracted from paraffin-embedded specimen of the splenic hamartomas
